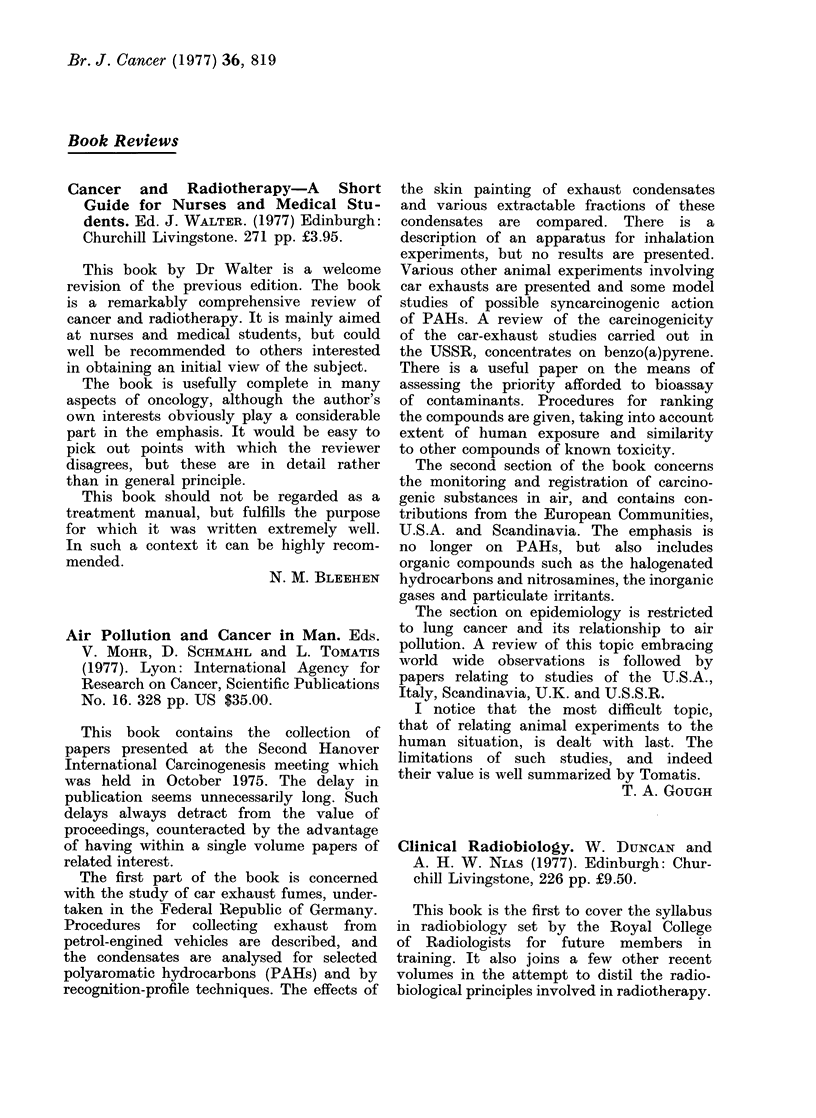# Cancer and Radiotherapy—A Short Guide for Nurses and Medical Students

**Published:** 1977-12

**Authors:** N. M. Bleehen


*Br. J. Cancer* (1977) **36**, 819

### *Book Reviews*

**Cancer and Radiotherapy—A Short Guide for Nurses and Medical Students.** Ed. J. Walter. (1977) Edinburgh: Churchill Livingstone. 271 pp. £3.95.

This book by Dr Walter is a welcome revision of the previous edition. The book is a remarkably comprehensive review of cancer and radiotherapy. It is mainly aimed at nurses and medical students, but could well be recommended to others interested in obtaining an initial view of the subject.

The book is usefully complete in many aspects of oncology, although the author's own interests obviously play a considerable part in the emphasis. It would be easy to pick out points with which the reviewer disagrees, but these are in detail rather than in general principle.

This book should not be regarded as a treatment manual, but fulfills the purpose for which it was written extremely well. In such a context it can be highly recommended.

N. M. Bleehen